# Shaoyao Decoction Inhibits Inflammation and Improves Intestinal Barrier Function in Mice With Dextran Sulfate Sodium-Induced Colitis

**DOI:** 10.3389/fphar.2021.524287

**Published:** 2021-04-20

**Authors:** Honggang Chi, Dan Wang, Mengting Chen, Jiantao Lin, Shuhua Zhang, Fengyan Yu, Jun Zhou, Xuebao Zheng, Ying Zou

**Affiliations:** ^1^ Department of Traditional Chinese Medicine, The Second Clinical Medical College, Guangdong Medical University, Dongguan, China; ^2^ Department of Traditional Chinese Medicine, The First Dongguan Affiliated Hospital of Guangdong Medical University, Dongguan, China; ^3^ Department of Pharmacology, Guangdong Medical University, Dongguan, China; ^4^ The Second Clinical Medical College, Guangdong Medical University, Dongguan, China; ^5^ Department of Pathology, Nanfang Hospital, Southern Medical University, Guangzhou, China; ^6^ Mathematical Engineering Academy of Chinese Medicine, Guangzhou University of Chinese Medicine, Dongguan, China; ^7^ Department of Traditional Chinese Medicine, Dongguan Liaobu Hospital, Dongguan, China

**Keywords:** inflammatory bowel disease, colitis, intestinal barrier function, shaoyao decoction, traditional Chinese medicine, mucus barrier, epithelial barrier

## Abstract

Shaoyao decoction (SYD), a classical traditional Chinese medicine formula, is effective for the treatment of inflammatory bowel disease (IBD). This study was designed to investigate the therapeutic effects of SYD on IBD and possible mechanisms. Dextran sulfate sodium (DSS, 3.5%) was used to induce colitis in C57BL/6 mice. Disease phenotypes were investigated based on disease activity index (DAI), colon length, and microscopic and macroscopic scores. Additionally, the presence of proinflammatory cytokines, immune cell infiltrates, intestinal cell proliferation, apoptosis, epithelial permeability, signal transducer and activator of transcription 3 (STAT3), and nuclear factor-κB (NF-κB) signaling, as well as the intestinal mucosal barrier function, were investigated. The administration of SYD significantly ameliorated the clinical signs, suppressed the levels of proinflammatory cytokines, and reduced immune cell infiltrates into colonic tissues of DSS-induced colitis model mice. SYD also significantly reduced the DSS-induced activation of STAT3 and NF-κB signaling. Furthermore, SYD promoted epithelial integrity by regulating epithelial cell apoptosis and epithelial permeability. Finally, we demonstrated that SYD protected the intestinal barrier function by significantly regulating the mucus layer genes *Muc1*, *Muc2*, *Muc4*, and *Tff3*, as well as the epithelial barrier genes *Z O -1* and *Occludin*. Our results indicate that SYD has a protective effect on DSS-induced colitis, which is attributable to its anti-inflammatory activity and intestinal barrier function-enhancing effects. These results provide valuable insights into the pharmacological actions of SYD for the treatment of IBD.

## Introduction

Inflammatory bowel disease (IBD), including ulcerative colitis (UC) and Crohn’s disease, is a group of chronic, relapsing, and remitting inflammatory disorders of the gastrointestinal tract characterized by fever, bloody diarrhea, abdominal cramping, and weight loss (Ivanov et al., 2009). Patients with IBD have a poor quality of life and are at an increased risk of developing colorectal cancer (colitis-associated colorectal cancer, CAC) compared to the general population (Neurath, 2014b). The etiology of IBD is complex and involves multiple factors, including genes, the host immune system, and environmental triggers, such as diet and the gut microbiota (He et al., 2019). Accumulating evidence suggests that an elevated inflammatory response and intestinal barrier dysfunction are central events in the pathogenesis of IBD (Neurath, 2014b). Currently, IBD therapy is directed toward dampening the inflammatory response in the gastrointestinal tract to alleviate symptoms and involves the use of immunomodulators, anti-tumor necrosis factor-alpha (TNF-α) antibodies, 5-aminosalicylic acid agents, and antimicrobials (Baumgart and Sandborn, 2012; Cheifetz et al., 2017). Although the currently used treatment methods are generally considered effective, not all patients achieve sustained remission and treatment is usually limited by adverse reactions (Quezada et al., 2018). Thus, alternative medicines, such as traditional Chinese medicine formulas and natural food substances, are desirable because of their efficacy and safety.

Traditional Chinese medicine, which is based on a multicomponent, multitarget approach, has a long history of widespread clinical use in China. Shaoyao decoction (SYD) is a traditional herbal medicine composed of Paeoniae Radix Alba (Paeonia lactiflora Pall.), Angelicae Sinensis Radix (Angelica sinensis (Oliv.) Diels), Coptidis Rhizoma (Coptis chinensis Franch.), Arecae Semen (Areca catechu L.), Aucklandiae Radix (Aucklandia costus Falc.), Rhei Radix et Rhizoma (Rheum palmatum L., R. tanguticum Maxim. ex Balf. or R. officinale Bail), Scutellariae Radix (Scutellaria baicalensis Georgi), Cinnamomi Cortex (Cinnamomum verum J.Presl), and Glycyrrhizae Radix et Rhizoma (Glycyrrhiza uralensis Fisch., Glycyrrhiza inflata Bat. or Glycyrrhiza glabra L.). SYD has been widely used to treat various diseases associated with damp-heat syndrome in the intestines and is effective for the treatment of IBD (Chen, 2014). Recent studies have shown that it exhibits a variety of pharmacological activities, including anti-inflammation and anticancer effects (Lin et al., 2014; Wang et al., 2019). Although studies have demonstrated that SYD is effective in ameliorating the major manifestations of IBD (Zhong et al., 2019), the associated mechanism of action needs to be explored further. Therefore, the present study was designed to investigate the anti-inflammatory and protective effects, with respect to intestinal barrier function, of SYD in a dextran sulfate sodium (DSS)-induced colitis model.

## Materials and Methods

### Animals

Sex-and age-matched C57BL/6 mice (8–12 weeks old) were used in this study. They were purchased from Sun Yat-Sen University (Guangzhou, China). All mice were housed in a vivarium and maintained with a specific pathogen-free health status, in individually ventilated cages. All studies using mice followed protocols approved by the Animal Care and Use Committee of Guangdong Medical University.

### Composition and Preparation of SYD

SYD was composed of Paeoniae Radix Alba (Paeonia lactiflora Pall.) (30 g), Angelicae Sinensis Radix (Angelica sinensis (Oliv.) Diels) (15 g), Coptidis Rhizoma (Coptis chinensis Franch.), (15 g), Arecae Semen (Areca catechu L.), (6 g), Aucklandiae Radix (Aucklandia costus Falc.), (6 g), Rhei Radix et Rhizoma (Rheum palmatum L., R. tanguticum Maxim. ex Balf. or R. officinale Bail) (9 g), Scutellariae Radix (Scutellaria baicalensis Georgi) (15 g), Cinnamomi Cortex (Cinnamomum verum J.Presl) (5 g), and Glycyrrhizae Radix et Rhizoma (Glycyrrhiza uralensis Fisch., Glycyrrhiza inflata Bat. or Glycyrrhiza glabra L.) (6 g). All these herbal materials were purchased from the Tongren Tang drug store (Dongguan, China) and were accredited by a pharmacologist, Professor Jiantao Lin. To prepare this formula, a total of 107 g of mixed SYD raw herbs were soaked in distilled water for 30 min and boiled in 10 volumes of water (v/w) for 1 h to yield a final volume 100 ml. The supernatant was then filtered with eight layers of surgical gauzes. Herb residues were again soaked in 600 ml water, boiled for 1 h, and filtered again. The two filtrates were mixed and vacuum-dried to obtain the powdered SYD extract. Finally, the SYD extract was dissolved in water to obtain SYD oral solution with the concentration of 1 g/mL crude drug, stored in the refrigerator at 4°C before intragastric administration to mice.

### High-Performance Lipid Chromatography (HPLC) Analysis

The chemical profile of SYD was determined using HPLC analysis. Standard chemicals (paeoniflorin and baicalin) were purchased from the National Institute for the Control of Pharmaceutical and Biological Products (Beijing, China). HPLC profiling was performed using an Agilent 1,200 liquid chromatography system (Agilent Technologies, Santa Clara, CA, United States) with a quaternary gradient pump, a column oven, an autosampler, online degasser, and diode-array detector. The separation was performed using an Eclipse Plus C18 column (4.6 × 150 mm, 5 μm) with a mobile phase composed of acetonitrile-phosphoric acid (0.05%; 15:85), a detection wavelength of 240 nm, a flow rate of 0.8 ml/min, and a column temperature of 25°C.

### Models of Experimental Colitis and SYD Treatment

After 1 week of acclimatization, the mice were randomly divided into the following three groups (n = 6): control, DSS and DSS + SYD groups. In all groups except the control group, colitis was induced by adding 3.5% DSS (molecular weight: 36,000–50,000, MP Biomedicals, Aurora, OH, United States) to the drinking water of C57BL/6 mice for 7 consecutive days, whereas the control group received regular drinking water. SYD (17.8 g/kg, raw herbs) was orally administered by gavage once daily from day 8 to day 15 until the termination of the experiment.

The dosage of SYD was determined according to the clinical equivalent dose of experimental mice. The clinical dosage of SYD for the human is 107 g (the total raw materials)/day. Equivalently, the dosage for mice is 17.8 g/kg/day calculated by the formula that converts humans’ dosage into that of mice according to the equivalent body surface area in accordance with the Chinese Medicine Pharmacology Research Technology.

### Assessment of Disease Activity Index (DAI)

Severity was assessed daily by measuring clinical parameters, such as weight loss, rectal bleeding, and stool consistency (Neufert et al., 2007). Disease activity index (DAI) was determined by combining scores of 1) weight loss, 2) rectal bleeding, and 3) diarrhea scores, divided by 3. Each score was determined as follows: weight change was calculated as the percent difference in weight relative to the original body weight (0: <1%; 1: 1–5%; 2:5–10%; 3:10–15%, 4:>15%). Rectal bleeding was scored as follows: 0 = no bleeding, 1 = no visible blood but a change in stool color, 2 = small area of visible blood, 3 = large area be of visible blood, and 4 = gross blood on most stool. Diarrhea scores were determined as follows: 0 = stool was firm, 1 = stool was soft but could be picked up, 2 = stool could not be picked up but had shape, 3 = stool could not be picked up and had no shape, and 4 = stool was watery. At the end of the experiment (day 15), the entire colon, from the cecum to anus, was removed and colon length was measured using a millimeter ruler.

### Histological Observation and Scoring

For histological examination, the colons were fixed in 10% paraformaldehyde, embedded in paraffin, and sliced into 5 µm-thick sections. Sections were stained with hematoxylin and eosin (H&E) and histological evaluation was performed in a blinded manner using a modified validated scoring system described previously (Zou et al., 2016). The scores were as follows: 0 = normal, 1 = moderate mucosal inflammation without ulcer, 2 = severe mucosal inflammation with ulcer (<1 mm) or no ulcer, 3 = severe mucosal inflammation with ulcer (1–3 mm), and 4 = severe mucosal inflammation with ulcer (>3 mm).

### Macroscopic Scoring

The distal colon was removed, opened longitudinally, and gently rinsed with ice-cold phosphate buffer (pH 7.4). The samples were assessed by an investigator blinded to the project, and the criteria for evaluating macroscopic damage to the colon were as follows (Bell et al., 1995): 0 = normal, 1 = hyperemia without ulcers, 2 = linear ulcer with no significant inflammation, 3 = wide ulcers and necrosis and/or adhesions, and 4 = megacolon and/or stenosis and/or perforation.

### Myeloperoxidase (MPO) Activity

Assessment of MPO activity in the colon tissue was performed using a kit as described by the manufacturer (Nanjing Jiancheng Bioengineering Institute, Nanjing, China). The MPO activity was expressed in U/mg protein.

### Immunohistochemistry

Colonic tissues were fixed in 10% buffered neutral formalin, dehydrated, and embedded in paraffin. Sections (5 µm-thick) were deparaffinized and rehydrated, subjected to microwave antigen retrieval, and incubated with primary antibodies against Ki67 (Dako, Agilent, Santa Clara, CA), p-signal transducer and activator of transcription 3 (STAT3), p-p65, zonula occludens-1 (ZO-1), Occluding, mucin 1 (Muc1) and mucin 4 (Muc4), cluster of differentiation (CD) 3, and CD11 b (Bioss Antibodies, Woburn, MA, United States) overnight at 4°C. The tissues were further incubated with biotinylated secondary antibodies (Vector Labs, Burlingame, CA, United States) and streptavidin horseradish peroxidase with diaminobenzidine (Vector Labs).

### Enzyme-Linked Immunosorbent Assay (ELISA)

Protein levels were determined by ELISA using kits for TNF-α, interleukin-1β (IL-1β) (R&D Systems, Minneapolis, MN, US) and IL-6 (BD Biosciences, San Diego, CA, United States) according to the manufacturer’s protocols.

### RNA Extraction and Real-Time Polymerase Chain Reaction (PCR)

Total RNA was extracted from mouse colon tissue using TRIzol reagent (Invitrogen, Carlsbad, CA, United States) following the manufacturer’s instructions. The cDNA was synthesized via the reverse transcription of 0.5 µg of RNA samples using RevertAid First Strand cDNA Synthesis Kit (Thermo Fisher Scientific, Waltham, MA, United States), according to the manufacturer’s protocols. PCR was performed using an ABI 7500 Real-Time PCR System (Applied Biosystems, Foster City, CA, United States) and SYBR Green Realtime PCR Master Mix (Toyobo, Osaka, Japan). Quantization was performed by normalizing expression to the level of the endogenous control *GAPDH*. Primers and probes for *TNF-*α, *IL-6*, *IL-1*β, *Muc1*, *Muc2*, and *Muc4*, and trefoil factor 3 (*Tff3*) have been described elsewhere (Wang et al., 2004; Lv et al., 2015; Horibe et al., 2016; Bibi et al., 2017).

### Western Blot Analysis

The colonic mucosa was homogenized in cold radioimmunoprecipitation assay buffer containing a protease inhibitor cocktail (Roche Diagnostics, Basel, Switzerland). The protein concentration was measured using the Pierce bicinchoninic acid protein assay kit (Thermo Fisher Scientific, Indianapolis, IN, United States). After boiling the samples for 10 min, total protein (50 µg per lane) was separated by sodium dodecyl sulfate-polyacrylamide gel electrophoresis. Bands were transferred to a polyvinylidene difluoride membrane (Millipore, Burlington, MA, United States), blocked, and probed with antibodies against p-STAT3, STAT3, p-p65, p65, ZO-1, and Occludin (1:1,000, Cell Signaling Technology, Danvers, MA, United States). β-Actin (1:2,000, Santa Cruz, Dallas, TX, United States) was used as an internal control. After overnight incubation at 4°C, the blots were incubated with the secondary antibody for 1–2 h at room temperature. Images of the western blots were analyzed using an LI-COR Odyssey Infrared Imaging System (Lincoln, NE, United States). To perform densitometric analyses, ImageJ and Image Studio Lite V4.0 were used.

### Apoptosis Assay

The terminal deoxynucleotidyl transferase dUTP nick end labeling (TUNEL) assay was performed using the Apop Tag Kit (Millipore, Billerica, MA, United States), according to the manufacturer’s protocol. The percentage of apoptotic cells in the colon tissue in each section was counted in 10 different microscopic fields.

### Intestinal Permeability Assay

An *in vivo* permeability assay was performed using the fluorescein isothiocyanate (FITC)-labeled dextran method. Briefly, food and water were withdrawn for 4 h and mice were gavaged with a permeability tracer (0.6 mg/g body weight of FITC-labeled dextran, MW 4000; Sigma-Aldrich, St. Louis, MI, United States). Serum was collected 4 h later and the fluorescence intensity of each sample was measured using a fluorescence spectrophotometer (excitation, 492 nm; emission, 525 nm; CytoFluor 2,300 nm). The concentration of FITC-dextran in sera was determined using the FITC-dextran standard curve.

### Statistical Analysis

Statistical analysis was performed using the SPSS 16.0 (IMB, Armonk, NY, United States) statistical software and data are expressed as the mean ± SEM. For multiple groups, nonparametric Kruskal-Wallis or parametric one-way ANOVA was used. *p* values <0.05 represent statistically significant differences.

## Results

### Quality Control Analysis of SYD

**FIGURE 1:**
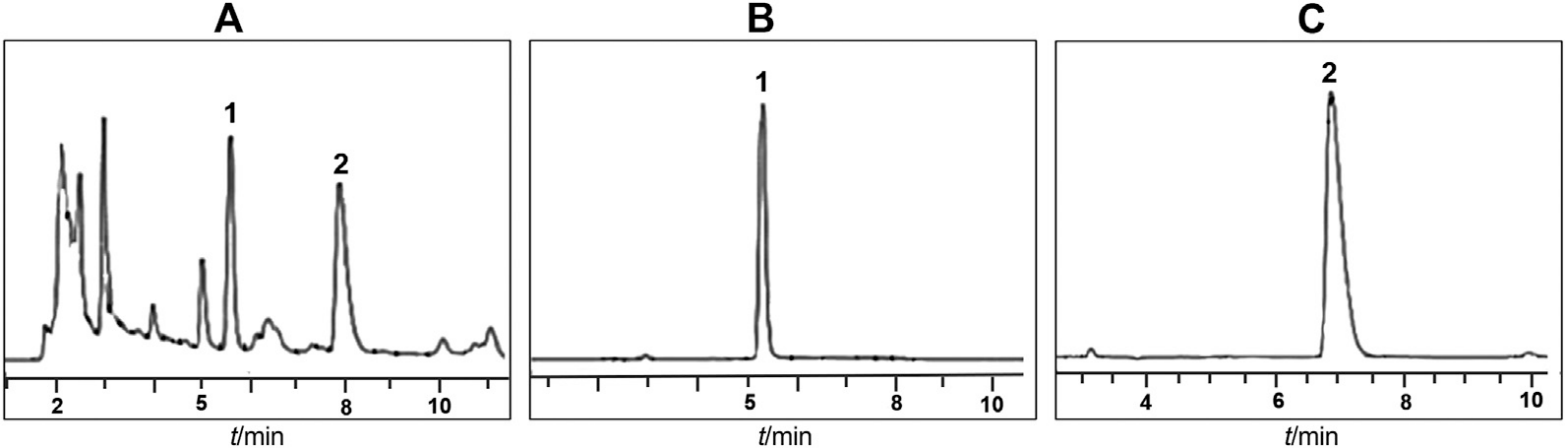
Identification of the chemical composition of SYD **(A)** HPLC profile of SYD sample **(B, C)** HPLC profile of standards: paeoniflorin and baicalin.

The chemical fingerprints of SYD were analyzed using HPLC to identify the chemicals contained in SYD. Two major bioactive components, paeoniflorin and baicalin, were identified using standard reference compounds. The concentrations of paeoniflorin and baicalin were 0.07 mg/g and 3.75 mg/g, respectively (Figures 1A–C).

### SYD Attenuates DSS-Induced Colitis

**FIGURE 2:**
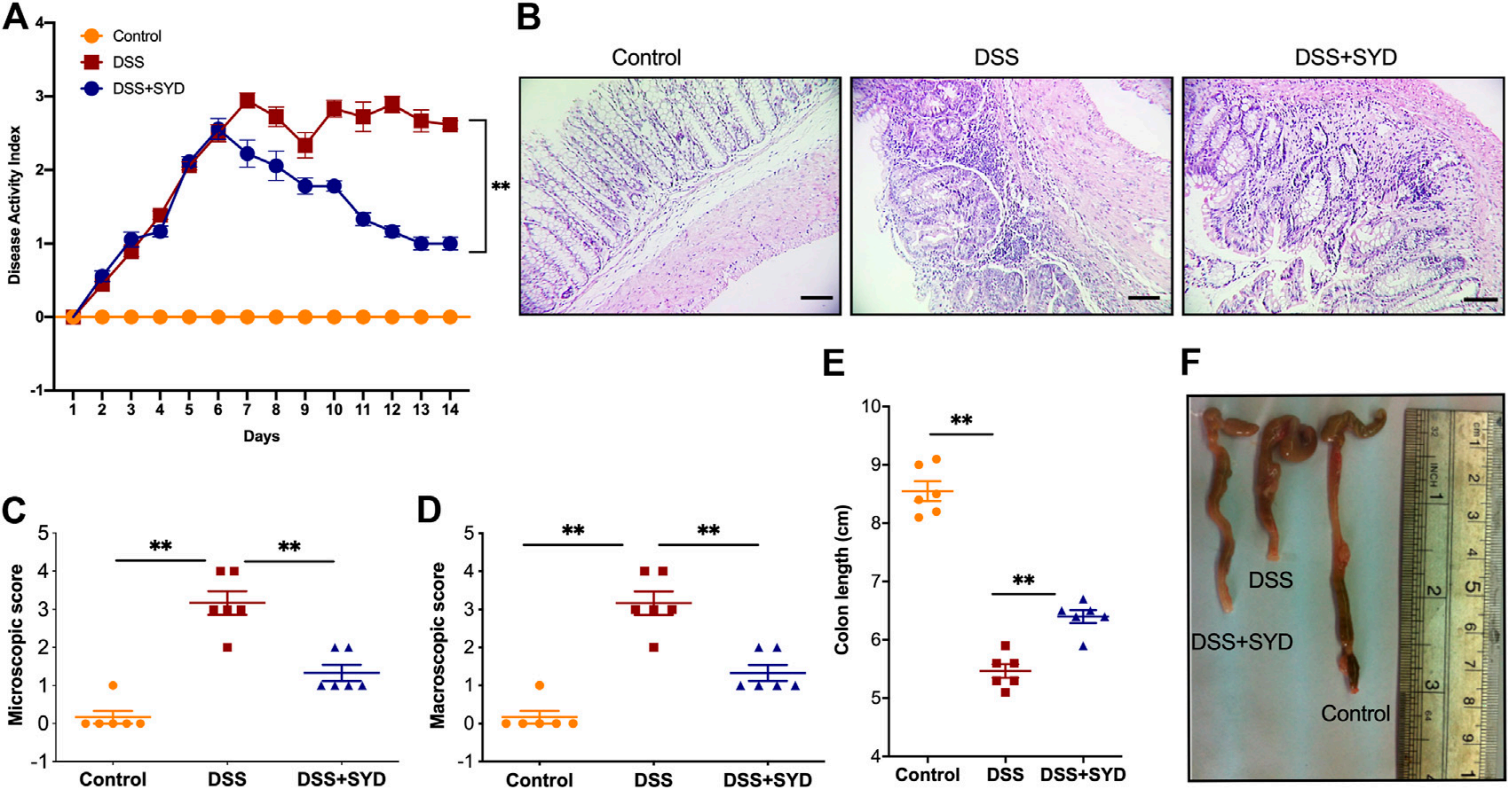
SYD protects mice from DSS-induced colitis **(A)** DAI scores were calculated **(B)** Serial sections of colon tissues were stained with hematoxylin and eosin (H&E). Scale bars = 100 μm **(C, D)** Microscopic and macroscopic score measurement **(E, F)** Colon length measurement. Data are presented as the mean ± SEM, n = 6 per group. *p < 0.05, **p < 0.01.

The therapeutic potential of SYD in colitis disease progression was investigated using a mouse model of DSS-induced experimental colitis. The mice were monitored daily for clinical signs of colitis, including body weight loss, rectal bleeding, and diarrhea. The DAI score increased significantly after DSS intake, whereas it was significantly reduced after SYD administration compared to those with DSS treatment (Figure 2A). Histological analysis showed progressive increases in inflammation, ulceration, edema, and hyperplastic proliferation of epithelial cells in the colons of mice after DSS administration. However, SYD-treated mice exhibited less morphological damage with largely preserved epithelial architecture and nearly intact intestinal crypts (Figure 2B). Similarly, SYD significantly decreased microscopic and macroscopic colon damage scores compared to those in the colitis mice (Figure 2C,D). Colon shortening, the marker of inflammation, was improved by SYD (Figures 2E,F). Collectively, these results suggested that SYD successfully ameliorated DSS-induced colitis.

### SYD Suppresses DSS-Induced Inflammatory Markers and Inflammatory Cell Infiltration in the Colon

**FIGURE 3:**
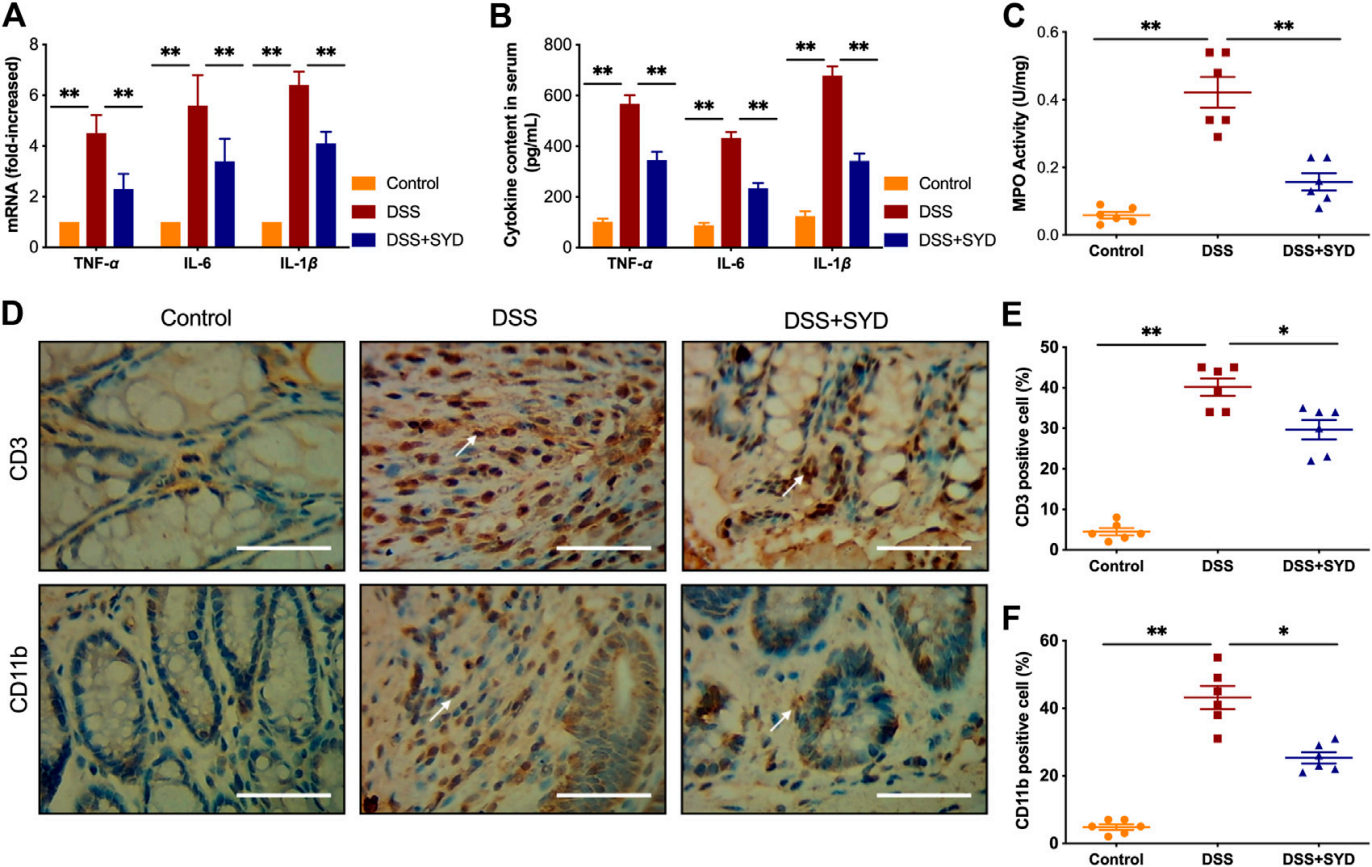
SYD inhibits proinflammatory mediators in colon tissues of DSS-induced colitis mice **(A)** The mRNA expression of *TNF-*α*, IL-6, and IL-1*β in colons was determined by real-time PCR **(B)** The protein levels of inflammation-related cytokines TNF-α, IL-6, and IL-1β in serum were determined by ELISA **(C)** Colonic myeloperoxidase (MPO) activity analysis **(D)** Immunohistochemical analysis of T cells (CD3^+^) and macrophages (CD11 b^+^) in colon sections. Scale bars = 100 μm **(E, F)** Quantification of CD3-positive and CD11 b-positive cell populations was performed on five fields of view per section. Data are presented as the mean ± SEM, n = 6 per group. *p < 0.05, **p < 0.01.

We next asked whether SYD regulates intestinal inflammatory responses and inflammatory cell infiltration in response to DSS-treatment. First, levels of proinflammatory cytokines, such as TNF-α, IL-6, and IL-1β, were measured by real time-PCR and ELISA. The levels of TNF-α, IL-6, and IL-1β in DSS-treated mice were significantly higher than those in control mice. However, the elevated levels of these cytokines in DSS-treated mice were significantly reduced by SYD treatment (Figures 3A,B).

We further examined the inflammatory activity of neutrophils using the MPO activity assay (Zaki et al., 2011). The colons of mice treated with DSS had substantially more colonic MPO expression than control mice. However, the administration of SYD significantly reduced MPO activity in DSS-treated mice (Figure 3C). To further determine the effects of SYD on intestinal immune cellularity, colon tissue sections were immunohistochemically stained to detect T cell (CD3^+^) and macrophage (CD11 b^+^) infiltration. Increased T cell and macrophage accumulation was observed in the colons of DSS-treated mice compared to that in control mice. SYD significantly reduced the recruitment of these inflammatory cells to the colon tissues (Figures 3D–F). In summary, these data further suggest a role for SYD in ameliorating intestinal inflammation during DSS-induced colitis.

### SYD Reduces DSS-Induced Activation of STAT3 and NF-κB Signaling

**FIGURE 4:**
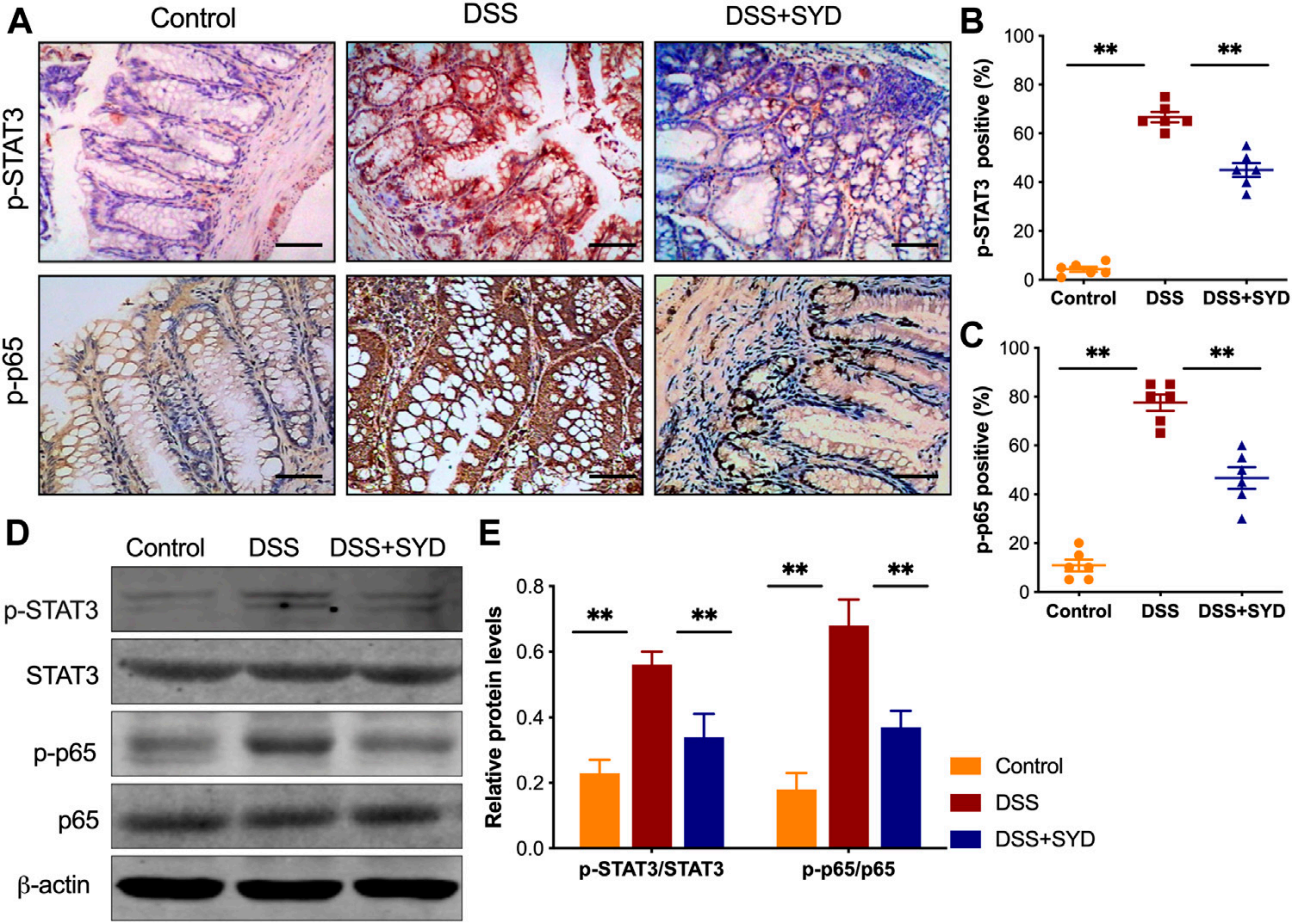
SYD inhibits the activation of STAT3 and NF-κB signaling in colon tissues of DSS-induced colitis mice **(A)** Immunohistochemistry of p-STAT3 and p-p65 in colon sections. Scale bars = 100 μm **(B, C)** Graphical representation of p-STAT3 and p-p65-positive cells in the mid-colon **(D, E)** The protein levels of p-STAT3, STAT3, p-p65, and p65 were measured by western blotting with relative densitometric values calculated as ratio of P- stat3/Stat3 and p-p65/P65. Each western blot is representative of three independent experiments. Data are presented as the mean ± SEM, n = 6 per group. *p < 0.05, **p < 0.01.

We next investigated whether SYD could inhibit the activation of STAT3 and nuclear factor- κB (NF-κB) signaling pathways which are known to regulate the transcription of proinflammatory genes (Atreya et al., 2008; Garrett et al., 2010). We first analyzed the phosphorylation level of STAT3 and p65 by immunohistochemistry. The expression of p-STAT3 and p-p65 in the DSS group was significantly higher than that in the control group. However, SYD significantly inhibited the expression of p-STAT3 and p-p65 (Figures 4A–C). Western blot analysis revealed significantly increased expression of p-STAT3 and p-p65 in colon tissues from the DSS treated mice compared to that of control animals. Such elevated expression was significantly decreased after treatment with SYD (Figures 4D,E). Together these data revealed an essential effect of SYD on inhibition of the activation of STAT3 and NF-κB signaling pathways during IBD.

### SYD Protects Intestinal Epithelial Integrity in DSS-Induced Colitis

**FIGURE 5:**
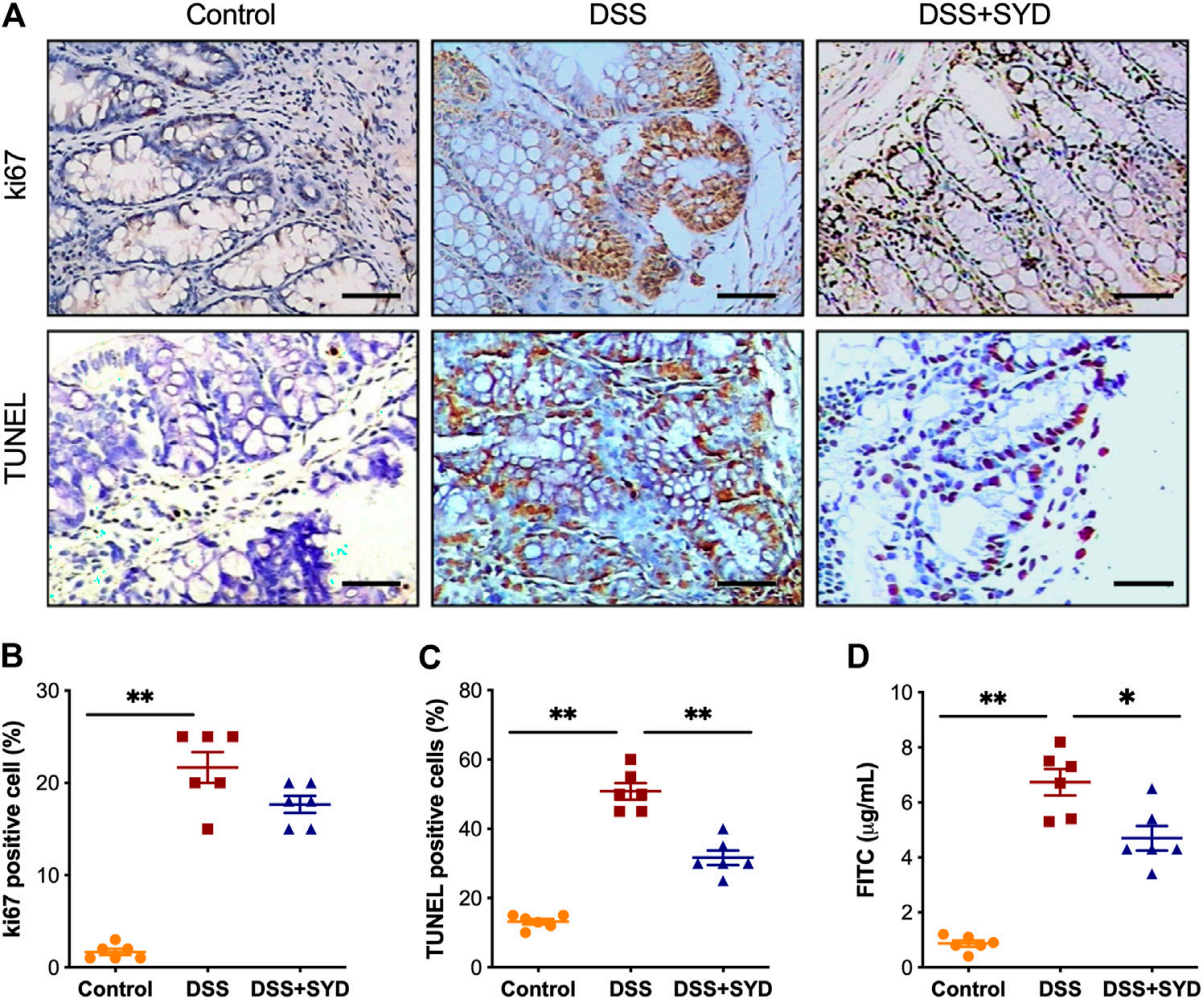
SYD regulates the proliferation and apoptosis of colonic enterocytes and intestinal epithelial permeability in DSS-induced colitis mice **(A)** Immunohistochemical analysis of proliferating cells detected based on Ki67 expression in colon sections and TUNEL assays for apoptosis. Scale bars = 100 μm **(B)** Percentage of Ki67-positive cells **(C)** Percentage of TUNEL-positive cells **(D)** Mice received oral gavage of FITC-dextran (0.6 mg/g), and serum FITC-dextran concentrations were determined 4 h later. Data are presented as the mean ± SEM, n = 6 per group. *p < 0.05, **p < 0.01.

To address whether SYD protects epithelial barrier integrity in DSS-induced colitis mice, the expression of the proliferation marker Ki67 was investigated through the immunohistochemical analysis of colonic Swiss-roll sections. The proportion of epithelial cells immunoreactive for Ki67 was significantly higher in colons from DSS-treated mice than in control mice. However, in mice treated with SYD, there was no significant change in Ki67 immunoreactivity than in DSS-treated controls (Figures 5A,B). We also investigated the presence of apoptotic cells in the colon after SYD treatment. The number of TUNEL-positive cells, comprising cells that have undergone both apoptosis and necrosis, was higher among colon epithelial cells obtained from DSS-treated mice than among colon epithelial cells obtained from control mice. By contrast, the number of TUNEL-positive cells was reduced by SYD treatment (Figure 5C).

Additionally, we investigated epithelial permeability by orally administering FITC-dextran to mice at the end of the experiment. We found that the serum level of FITC-dextran was significantly higher in DSS-treated mice than in control mice; however, it was reduced by SYD treatment (Figure 5D). Taken together, these data suggested that SYD protects intestinal epithelial integrity during intestinal inflammation.

### SYD Improves Colonic Mucus Barrier Function in DSS-Induced Colitis

**FIGURE 6:**
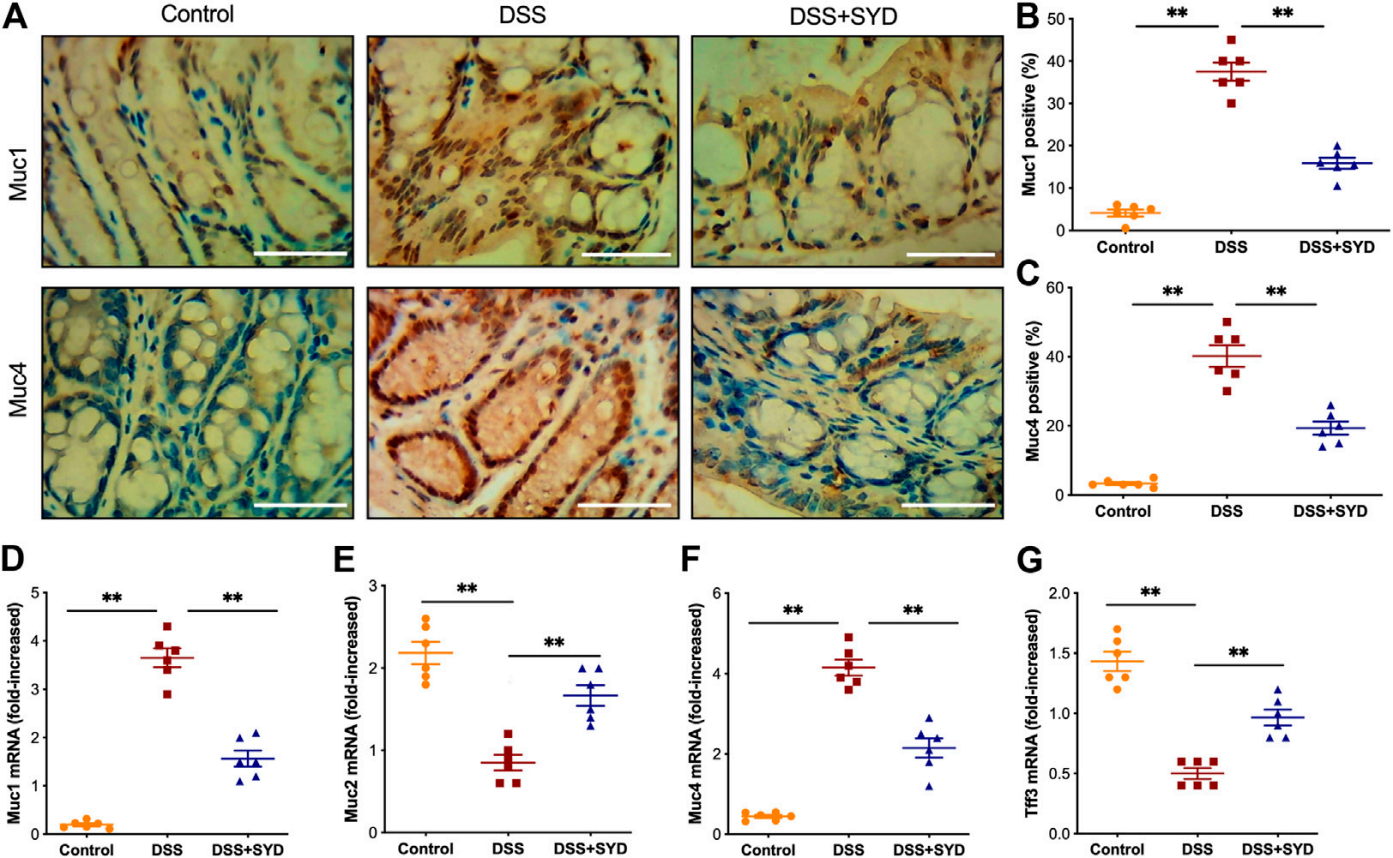
SYD improves colonic mucus barrier function in DSS-treated mice **(A)** Immunohistochemistry of Muc1 and Muc4 in colon sections. Scale bars = 100 μm **(B, C)** Graphical representation of the percentage of Muc1 and Muc4 in mid-colon **(D–G)** Expression of *Muc1, Muc2, Muc4,* and *Tff3* was evaluated by real-time PCR. Data are presented as the mean ± SEM, n = 6 per group. *p < 0.05, **p < 0.01.

To investigate the effects of SYD on the colonic mucus barrier in DSS-treated mice, we first performed immunohistochemistry analysis to determine the expression of Muc1 and Muc4. We found higher levels of Muc1 and Muc4 in the colons of DSS-treated mice than in control mice. However, these levels were reduced after the administration of SYD (Figures 6A–C). We next quantified the mRNA levels of *Muc1*, *Muc2*, *Muc4*, and *Tff3* using real-time PCR. The mRNA expression of *Muc1* and *Muc4* was lower in the colons of SYD-treated mice than in those of mice treated with DSS (Figures 6D,F). However, colonic *Muc2* and *Tff3* mRNA levels were significantly decreased in the colons of DSS-treated mice, whereas they were significantly increased in mice treated with SYD (Figures 6E,G). In summary, these results indicated that SYD has a significant effect on improving mucus barrier function.

### SYD has a Protective Effect on Epithelial Barrier Function in DSS-Induced Colitis

**FIGURE 7:**
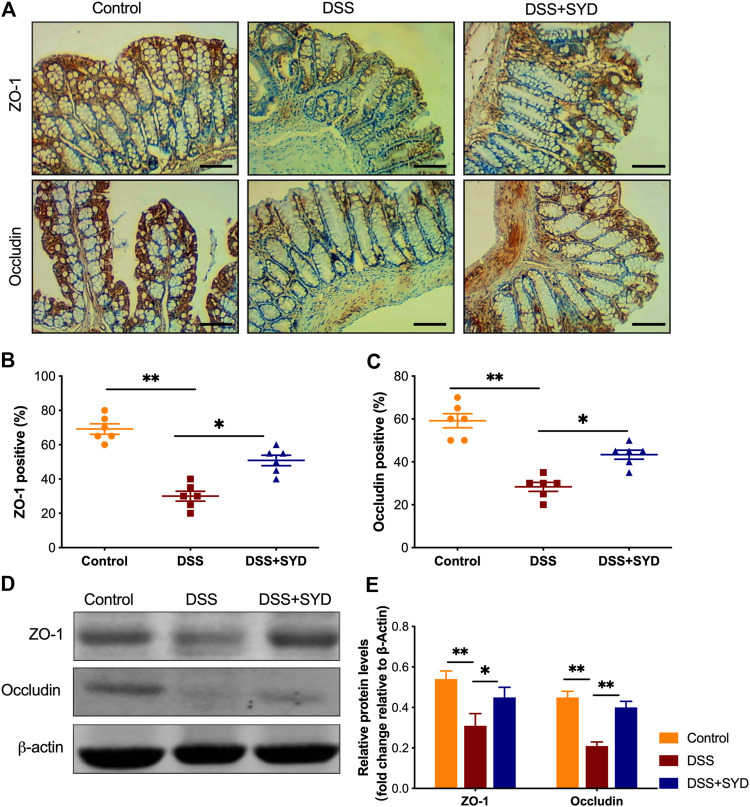
SYD promotes intestinal epithelial barrier function in DSS-induced colitis mice **(A)** Immunohistochemical analysis of tight junction (TJ) proteins ZO-1 and Occludin in colon sections Scale bars = 100 μm **(B, C)** Graphical representation of the percentage of ZO-1 and Occludin positive cells in the mid-colon **(D, E)** Immunoblot analysis of the expression of ZO-1 and Occludin in colon mucosa. Quantitative analysis of ZO-1 and Occludin normalized to β-actin. Each western blot is representative of three independent experiments. Data are presented as the mean ± SEM, n = 6 per group. *p < 0.05, **p < 0.01.

To study the role of SYD on the epithelial barrier function, we measured the expression levels of tight junction (TJ) proteins, including ZO-1 and Occludin. Immunohistochemistry analysis showed that the expression of ZO-1 and Occludin was significantly decreased after DSS exposure but increased after SYD treatment (Figures 7A–C). Moreover, western blot analysis revealed that SYD significantly increased the protein levels of ZO-1 and Occludin (Figures 7D,E). These findings indicated that SYD maintains intestinal epithelial barrier function by upregulating the expression of TJ proteins.

## Discussion

An uncontrolled mucosal inflammation response with impaired intestinal barrier function is a hallmark of the pathogenesis of IBD (Hing et al., 2013). SYD possesses anti-inflammatory and anticancer effects in IBD and colorectal cancer (Chen, 2014; Lin et al., 2014; Wang et al., 2019); however, how SYD modulates gut homeostasis and the pharmacological mechanisms underlying its effect on IBD remain unclear. Here, we report for the first time that the administration of SYD effectively ameliorates DSS-induced colitis and that the protective effect is associated with anti-inflammatory activity and the maintenance of intestinal barrier function. This indicates that SYD might be a potent, safe, and economical option for the treatment of IBD.

To investigate the protective effects of SYD in IBD, we used a DSS-induced colitis model, which has similar disease signs to human colitis (Okayasu et al., 1990). We demonstrate for the first time that oral administration of SYD (17.8 g/kg) effectively attenuates signs of DSS-induced colitis, including body weight loss, rectal bleeding, diarrhea, and decreased DAI. Furthermore, colonic pathological damage and microscopic and macroscopic scores, and colon shortening in mice were significantly ameliorated after 7 days of SYD treatment, suggesting a protective effect of SYD on experimental colitis.

Multiple cytokines have been implicated in the initiation, mediation, perpetuation, progression, and development of IBD (Neurath, 2014a). Increased levels of proinflammatory cytokines, such as TNF-α, IL-6, and IL-1β, are detected in mice and patients with IBD and correlate with the severity of inflammation (Sartor, 1994). Excessive levels of proinflammatory cytokines can damage the epithelial barrier and affect gut homeostasis (Kaser et al., 2010). A recent study reported that SYD decreases the serum levels of cytokines IL-1β, IL-6, and TNF-α in azoxymethane/dextran sulfate sodium-induced CAC model mice (Lin et al., 2014). Our study provides evidence that SYD reduces the levels of these proinflammatory cytokines both in the colon and serum of mice with DSS-induced colitis. The measurement of MPO activity can indirectly reflect the strength of neutrophil infiltration; thus, it can be used as a quantitative index of inflammation in colitis (Krawisz et al., 1984). Indeed, immune cells, including neutrophils, T cells, macrophages, and dendritic cells, in the inflamed gut, together with proinflammatory cytokines, are important mediators of the response to inflammation during the development of colitis (Zhou et al., 2019). In our study, SYD successfully inhibited the enhanced MPO activity in the colon of DSS-induced colitis mice. Furthermore, our study showed that the infiltration of T cells and macrophages increased in DSS-treated mice, whereas SYD successfully decreased the infiltration of T cells and macrophages into colon tissues. Overall, our findings showed an anti-inflammatory effect of SYD on DSS-induced colitis.

We further investigated the molecular mechanism underlying the SYD-mediated suppression of colonic inflammation during IBD development. NF-κB is a critical proinflammatory transcription factor that plays a crucial role in inflammation by inducing the transcription of pro-inflammatory genes (Tak and Firestein, 2001). Proinflammatory cytokines, including TNF-α, IL-6, and IL-1β, and the induction of proinflammatory enzymes, cyclooxygenase 2, and inducible nitric oxide synthase in the colon tissues are transcriptionally regulated by NF-κB, indicating its role in the severity of intestinal inflammation (Rogler et al., 1998; Qian et al., 2015). Meanwhile, recent studies have revealed a critical role of STAT3 activation in IBD (Kasembeli et al., 2018; Ye et al., 2019). Constitutive activation of STAT3 in actively inflamed colons has been observed in IBD patients (Lovato et al., 2003; Mudter et al., 2005; Li et al., 2010). The levels of activated STAT3 in T-cells, epithelial cells and macrophages, have been shown to directly correlate with the severity of colitis (Musso et al., 2005; Li et al., 2010). Our study showed that SYD inhibited the activation of STAT3 and NF-κB signaling during IBD. These findings suggest that this treatment can attenuate the DSS-induced inflammatory response in the colon, at least partly, via the inhibition of both STAT3 and NF-κB signaling pathways.

Our results also demonstrated that the effects of SYD on colitis were related to an improvement in epithelial barrier integrity. A critical function of the intestinal mucosa is to form a barrier against microbiota and pathogens (Citi, 2018). Repeated damage to the intestinal epithelium accompanied by disruption of the intestinal barrier is a hallmark of IBD (Schoultz and Keita, 2019). Increased epithelial cell apoptosis, which compromises the mucosal barrier and increases mucosal permeability and reflects epithelial barrier dysfunction, is frequently observed in both experimental colitis and patients (Mankertz and Schulzke, 2007). The maintenance of epithelial homeostasis to ensure an effective intestinal barrier depends on the balance between cell proliferation and epithelial apoptosis. Colonic epithelial cell proliferation is an important feature of mucosal repair (Neurath, 2014b). Furthermore, the inhibition of apoptosis of intestinal epithelial cells can promote healing of the mucosa (Lee et al., 2020). In this study, we showed that SYD inhibited gut epithelial apoptosis and decreased mucosal permeability. However, the proliferation assay using Ki67 staining revealed no significant changes in SYD-treated mice. These results led us to postulate that SYD facilitates mucosal repair by inhibiting epithelial cell apoptosis, with no effect on proliferation.

The colonic mucosal barrier, which is the first physical barrier in intestinal innate immunity, consists of a mucus layer and epithelial cells, and the deterioration of this barrier is prominent in IBD patients (Podolsky and Isselbacher, 1984; Rhodes, 1996; Jager et al., 2013). In patients with UC and experimental colitis models, characterized by a thin mucin layer in association with goblet cell depletion, high expression of Muc1 and Muc4, and low expression of Muc2 and Tff3 have been reported (Longman et al., 2006; van der Post et al., 2019). Epithelial barrier dysfunction is a key feature of human IBD (Langer et al., 2019). The altered expression of TJ proteins, including ZO-1 and Occludin, plays a crucial role in regulating epithelial barrier function (Vivinus-Nebot et al., 2014). In this study, we found that Muc2 and Tff3 levels in the colon were enhanced after SYD treatment. However, the expression of Muc1 and Muc4 in the colon was decreased in response to SYD treatment. Furthermore, the expression of ZO-1 and Occludin in the colon was increased in the SYD group. Collectively, our results suggest that SYD enhances mucosal barrier function by not only promoting mucus secretion but also by restoring injured epithelial structures.

## Conclusion

In summary, our observations indicate the protective effect of SYD in a mouse model of DSS-induced colitis. This protective effect could be mediated by multiple processes, including the inhibition of inflammation, suppression of the stimulated STAT3 and NF-κB signaling pathways, and an improvement in intestinal barrier function in the colon. Our results demonstrated that SYD could be considered a safe, effective, and economical therapeutic alternative for the prevention and management of colitis.

## Data Availability

The data generated or analyzed during this study are available from the corresponding author upon reasonable request.
